# Enhanced photoluminescence from porous silicon nanowire arrays

**DOI:** 10.1186/1556-276X-8-277

**Published:** 2013-06-09

**Authors:** Chunqian Zhang, Chuanbo Li, Zhi Liu, Jun Zheng, Chunlai Xue, Yuhua Zuo, Buwen Cheng, Qiming Wang

**Affiliations:** 1 State Key Laboratory on Integrated Optoelectronics, Institute of Semiconductors, Chinese Academy of Sciences, Beijing 100083, China

**Keywords:** Si nanowire, Porous structure, PL, 78.55.-m, 78.67.Uh, 78.55.Mb

## Abstract

The enhanced room-temperature photoluminescence of porous Si nanowire arrays and its mechanism are investigated. Over 4 orders of magnitude enhancement of light intensity is observed by tuning their nanostructures and surface modification. It is concluded that the localized states related to Si-O bonds and self-trapped excitations in the nanoporous structures are attributed to the strong light emission.

## Background

The past decade has seen intense interest in nanoscale structures as these materials exhibit significantly different optical and electrical properties from their bulk materials [1-4]. Si, as one of the most conventional semiconductor materials, plays an important role in microelectronics [5-7]. Its application in integrated circuits has drastically changed the way we live. However, due to its indirect bandgap structure, the weak light emission from Si limits its application for future on-chip optical interconnection. Much effort has been devoted to overcome this limitation [8-10], and nanostructured materials are believed to be a candidate for light-emitting devices. There have been many reports discussing light emission and its mechanism from porous Si [11-13], Si sphere [14], and nanowire [3,15-20] structures. Several perspectives, such as quantum size effects [2], interfacial state [11,14], and radiative defects in SiO_*x*_[19,21] are used to explain their contribution on the strong photoluminescence (PL). However, there are only limited investigations on the enhancement of light emission. In this letter, we will discuss the ways to improve the PL properties of porous Si nanowire arrays. Over 4 orders of magnitude enhancement of PL intensity is observed at room temperature by engineering their nanostructures and chemically modifying their surfaces.

## Methods

Si nanowire arrays (Si NWAs) were prepared by metal-assisted chemical etching on p-Si(100) with the resistivity of 0.02 Ω cm. The Si wafers were firstly cleaned in acetone, ethanol, and diluted hydrofluoric acid (HF) solution to remove the organic contaminants and the native SiO_2_ layer. Ag particles were then formed in the solution of AgNO_3_ (0.06 M) and HF (5 M) for 10 min followed by the chemical etching of Si NWAs in the solution of HF (5 M) and H_2_O_2_ for 15 min. Ag catalysts were finally removed in concentrated HNO_3_. Si NWAs with different surface morphology were obtained by tuning the H_2_O_2_ concentration at 0.2, 0.5, 2, and 5 M. Scanning electron microscopy (SEM) and transmission electron microscopy (TEM) were utilized to investigate the surface morphology and the crystallinity of the Si nanowires. PL measurements were performed to investigate their optical property with LabRam HR 800 Raman instrumentation (Horiba Jobin Yvon) within the range of 500 to 1,000 nm using the 488-nm line of an Ar^+^ laser at a laser power of 2 mW.

## Results and discussion

**Figure 1:**
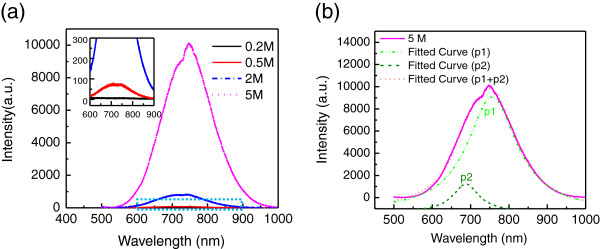
**Room-temperature PL spectra of Si NWAs prepared at different concentrations.** (**a**) PL spectrum of Si NWAs prepared at different H_2_O_2_ concentrations. Inset is the enlarged PL spectrum in the dotted square area. (**b**) The fitted PL spectrum of Si NWAs obtained at 5 M H_2_O_2_ concentration.

**Figure 2:**
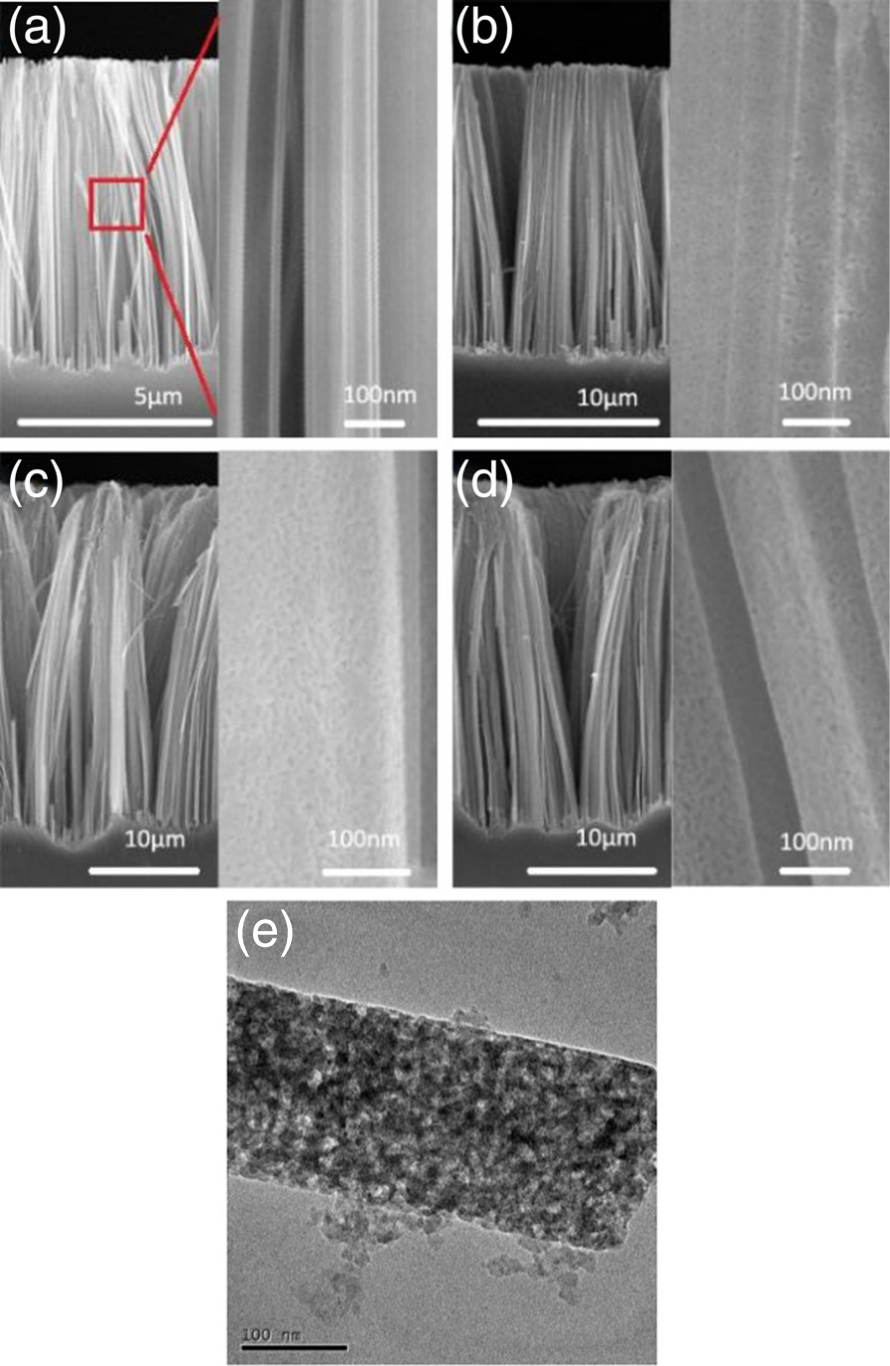
**SEM and TEM images of Si NWAs prepared at different H**_**2**_**O**_**2 **_**concentrations.** SEM images of Si NWAs prepared at different H_2_O_2_ concentrations: (**a**) 0.2, (**b**) 0.5, (**c**) 2, and (**d**) 5 M, and their enlarged images. The nanowires have diameters of 30 to 200 nm. (**e**) TEM image of porous Si NWAs prepared at 5 M H_2_O_2_ concentration.

Figure 1 shows the room-temperature PL spectra of Si NWAs prepared in different conditions. Clearly, with the increase of H_2_O_2_ concentration, the PL intensity increases greatly. Four orders of magnitude enhancement of light intensity is observed for the Si NWAs prepared at 5M H_2_O_2_ concentration compared to that obtained at 0.2 M H_2_O_2_ concentration, which only exhibits a very weak PL spectrum (as shown in the inset of Figure 1a). From the SEM images of Si NWAs in Figure 2, we find that at low H_2_O_2_ concentration (0.2 M), the NWAs have a smooth NW surface (Figure 2a) whereas at higher H_2_O_2_ concentration, they exhibit porous structures (Figure 2b,c,d,e). The porosity of NWAs increases with the increase of H_2_O_2_ concentration. This trend is consistent with that found in the PL intensity in Figure 1a, and it indicates that the PL enhancement is related to the surface nanostructures of Si NWAs.

All the PL emissions in Figure 1a exhibit similar broad peaks centered around 750 nm with a short-wavelength shoulder. They can be deconvoluted to two bands centered at 752 and 688 nm as shown in Figure 1b. The former (p1) is consistent with reports before [3], and it is believed to arise from the silicon nanostructure coated with a thin oxide layer. However, the weak PL peak located at 688 nm has not been discussed yet. It is 8 nm longer than that observed in [19,20]. This red shift may be due to the relatively big skeleton size (approximately 20 nm) of the porous NWA as shown in Figure 2d or from other emission mechanisms.

**Figure 3:**
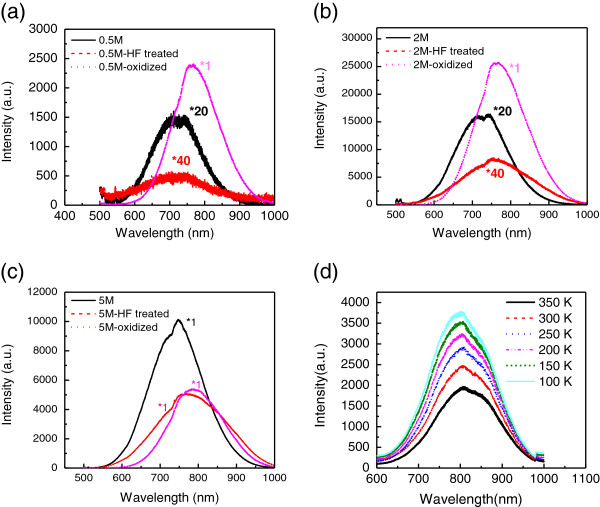
**PL spectra of pristine and treated Si NWA samples.** PL spectra of treated Si NWA samples prepared with H_2_O_2_ concentrations of (**a**) 0.5, (**b**) 2, and (**c**) 5 M at room temperature. The symbol ‘*’ denotes the multiplying factor relative to their original PL. (**d**) Temperature-dependent PL spectrum of oxidized Si NWAs obtained at 5 M H_2_O_2_ concentration.

To investigate the enhancement mechanism of light emission from the porous Si NWAs and confirm their emission origins, these samples are divided into two groups and processed with further treatment. For group 1, oxidization was performed at 1,000°C for 5 min to passivate the surface with Si-O bonds; in group 2, the Si NWAs were rinsed in diluted HF to remove the Si-O bonds on the surface. Figure 3 shows the PL spectra of pristine and treated NWA samples. Interestingly, for the samples with low porosity (those obtained at 0.2, 0.5, and 2 M H_2_O_2_ concentrations), oxidization treatments are always helpful to improve the PL intensity, and over 30 times enhancement is observed compared to their pristine ones. This is easily understood as the intense SiO_2_ surface can greatly reduce the nonradiative recombination and help the light emission. The maximum PL intensity comes from the oxidized Si NWAs prepared at 2 M H_2_O_2_ concentration, and a 2.5 × 10^4^ times enhancement is observed compared to that from Si NWAs prepared at 0.2 M (solid line in the inset of Figure 1a). However, for the NWAs obtained at 5 M H_2_O_2_ concentration, an opposite trend is observed. After oxidization, the PL intensity has a twofold decrease, and we attribute this to the reduction of effective light-emitting centers or interface state as the small-sized silicon skeleton is fully oxidized into SiO_2_. Even proper thermal oxidization helps the light emission from the Si NWAs; compared with the 4 orders of magnitude enhancement for the pristine samples as shown in Figure 1a, only 2 orders of magnitude enhancement is observed with the increase of H_2_O_2_ concentration for all oxidized Si NWAs. In our experiment, we find that the best PL intensity comes from the thermal treatment at 1,000°C for 5 min for the Si NWA sample prepared at 2M H_2_O_2_ concentration. Long-time thermal oxidization will induce the reduction of PL intensity as the small nanostructures are oxidized.

To our surprise, after oxidization, the PL peaks have a red shift for all the samples. The shift increases with the porosity of NWAs, and a maximum shift of 50 nm from 750 to 800 nm was observed for the sample prepared at 5 M H_2_O_2_ concentration. This phenomenon cannot be explained by the quantum confinement (QC) effect. According to QC theory, the bandgap should increase with the size decrease of the nanostructure by oxidization and lead to a blue shift. Moreover, their temperature-dependent PL spectrum also indicates that the light emission did not originate from the QC effect. As shown in Figure 3d, the intensity of PL increases with decreasing temperature, while the peak position remains stable. Apparently, the emission mechanism is also contradictive with the well-known Varshni formula in the QC that it will induce a blueshift with decreasing temperature. At the same time, the emission linewidth decreases with increasing temperature in porous Si NW arrays. This abnormal phenomenon has been explained by a multilevel model for light emission as discussed before [18].

Simultaneously, HF treatment on the Si NWAs always arouses the great decrease of intensity. We know that HF treatment removes the Si-O layer and introduces the Si-H bonds on the surface, which will impede the formation of new Si-O bonds, so light emission and its enhancement should be related to the Si-O-bonded nanostructure. The localized state related to Si-O bonds and self-trapped excitations in the nanoporous structures are the main origins of the light emission. With the increase of the porosity of Si NWAs at high H_2_O_2_ concentration, it offers more light-emitting centers and the PL intensity is greatly enhanced. From Figure 3a,b,c, it is found that the small shoulder in the short wavelength corresponding to the p2 peak disappears, and it agrees well with the discussion in [19].

## Conclusion

Si NWAs on Si substrates with different morphology were prepared by two-step metal-assisted chemical etching. With the increase of porosity, the light emission intensity increases. Surface treatment affects the intensity significantly, and oxidization substantially strengthens the intensity. The origin of the strong emission of Si NWAs is concluded to be from the localized state related to Si-O bonds and self-trapped excitations in the nanoporous structures.

## Competing interests

The authors declare that they have no competing interests.

## Authors’ contributions

CZ and CL designed the study and carried out the experiments. CZ, ZL, JZ, and CX performed the treatment of experimental data. CZ, CL, YZ, BC, and QW took part in the discussions of the results and prepared the manuscript initially. All authors read and approved the final manuscript.
